# Supplementary material for: Cosmetic Treatment Using Botulinum Toxin in the Oral and Maxillofacial Area: A Narrative Review of Esthetic Techniques
Source: Toxins (Basel). 2023 Jan 17;15(2):82. doi: 10.3390/toxins15020082 (PMC9964918; doi:10.3390/toxins15020082)
Supplement: Supplementary file 1 [file toxins-15-00082-s001.zip › toxins-2101528-supplementary.pdf]

Table S1. List of 27 consensus recommendation papers retrieved for the literature review. Three studies using abotulinumtoxinA were excluded from the review.

	Authors	Year	Title	BoNT type
**1**	Carruthers et al. [33]	2004	Consensus recommendations on the use of botulinum toxin type a in facial aesthetics	onabotulinumtoxinA
**2**	Carruthers et al. [34]	2008	Advances in facial rejuvenation: botulinum toxin type a, hyaluronic acid dermal fillers, and combination therapies-- consensus recommendations	onabotulinumtoxinA
**3**	Carruthers et al. [35]	2013	The convergence of medicine and neurotoxins: a focus on botulinum toxin type A and its application in aesthetic medicine-- a global, evidence-based botulinum toxin consensus education initiative: part II: incorporating botulinum toxin into aesthetic clinical practice	onabotulinumtoxinA
**4**	Fagien & Raspaldo [31]	2007	Facial rejuvenation with botulinum neurotoxin: an anatomical and experiential perspective	onabotulinumtoxinA
**5**	Raspaldo et al. [39]	2011	Upper- and mid-face anti-aging treatment and prevention using onabotulinumtoxin A: the 2010 multidisciplinary French consensus--part 1	onabotulinumtoxinA
**6**	Raspaldo et al. [38]	2011	Lower-face and neck antiaging treatment and prevention using onabotulinumtoxin A: the 2010 multidisciplinary French consensus–part 2	onabotulinumtoxinA
**7**	Raspaldo et al. [36]	2012	Global, 3-dimensional approach to natural rejuvenation: part 1 - recommendations for volume restoration and the periocular area	onabotulinumtoxinA
**8**	Gassia et al. [37]	2013	Global 3-dimensional approach to natural rejuvenation: recommendations for perioral, nose, and ear rejuvenation	onabotulinumtoxinA
**9**	Maas et al. [54]	2012	Current aesthetic use of abobotulinumtoxinA in clinical practice: an evidence-based consensus review	abobotulinumtoxinA &onabotulinumtoxinA
**10**	Imhof et al. [88]	2013	S1 guideline aesthetic botulinum toxin therapy	onabotulinumtoxinA
**11**	Lorenc et al. [87]	2013	Consensus panel's assessment and recommendations on the use of 3 botulinum toxin type A products in facial aesthetics	onabotulinumtoxinA
**12**	De Maio et al. [59]	2017	Facial assessment and injection guide for botulinum toxin and injectable Hyaluronic Acid fillers: focus on the upper face	onabotulinumtoxinA
**13**	De Maio et al. [60]	2017	Facial assessment and injection guide for botulinum toxin and injectable hyaluronic Acid fillers: focus on the midface	onabotulinumtoxinA
**14**	De Maio et al. [71]	2017	Facial assessment and injection guide for botulinum toxin and injectable Hyaluronic Acid fillers: focus on the lower face	onabotulinumtoxinA
**15**	Ahn et al. [27]	2013	Consensus recommendations on the aesthetic usage of botulinum toxin type A in Asians	Medytox
**16**	Yutskovskaya et al. [43]	2015	IncobotulinumtoxinA in aesthetics: Russian multidisciplinary expert consensus recommendations	incobotulinumtoxinA
**17**	Wu et al. [30]	2016	Consensus on current injectable treatment strategies in the Asian face	onabotulinumtoxinA
**18**	Kapoor et al. [29]	2017	Consensus recommendations for treatment strategies in Indians using botulinum toxin and	onabotulinumtoxinA

			Hyaluronic Acid fillers	
**19**	Sundaram et al. [83]	2016	Global aesthetics consensus: Botulinum toxin type A--evidence-based review, emerging concepts, and consensus recommendations for aesthetic use, including updates on complications	onabotulinumtoxinA
**20**	Sundaram et al. [84]	2016	Global aesthetics consensus: Hyaluronic Acid fillers and botulinum toxin type A-recommendations for combined treatment and optimizing outcomes in diverse patient populations	onabotulinumtoxinA
**21**	Sundaram et al. [28]	2016	Aesthetic applications of botulinum toxin A in Asians: an international, multidisciplinary, Pan-Asian consensus	incobotulinumtoxinA
**22**	Kaminer et al. [86]	2020	Re-examining the optimal use of neuromodulators and the changing landscape: a consensus panel update	onabotulinumtoxinA
**23**	Bertossi et al. [32]	2018	Italian consensus report on the aesthetic use of onabotulinum toxin A	onabotulinumtoxinA
**24**	Signorini et al. [85]	2022	OnabotulinumtoxinA from lines to facial reshaping: A new Italian consensus report	onabotulinumtoxinA
**25**	Ascher et al. [42]	2010	International consensus recommendations on the aesthetic usage of botulinum toxin type A (Speywood Unit)-Part I: upper facial wrinkles	abobotulinumtoxinA
**26**	Ascher et al. [40]	2010	International consensus recommendations on the aesthetic usage of botulinum toxin type A (Speywood Unit)-Part II: wrinkles on the middle and lower face, neck and chest	abobotulinumtoxinA
**27**	Kane et al. [41]	2010	Expanding the use of neurotoxins in facial aesthetics: a consensus panel's assessment and recommendations	abobotulinumtoxinA
